# When 2 become 1: Autistic simultaneity judgements about asynchronous audiovisual speech

**DOI:** 10.1177/17470218231197518

**Published:** 2023-09-20

**Authors:** Daniel Poole, Emma Gowen, Ellen Poliakoff, Anna Lambrechts, Luke A Jones

**Affiliations:** 1 School of Health Sciences, The University of Manchester, Manchester, UK; 2 Department of Psychology, University of Sheffield, Sheffield, UK; 3 Autism Research Group, City, University of London, London, UK

**Keywords:** Autism, timing, simultaneity judgement, audiovisual, drift-diffusion model, temporal binding window

## Abstract

It has been proposed that autistic people experience a temporal distortion whereby the temporal binding window of multisensory integration is extended. Research to date has focused on autistic children so whether these differences persist into adulthood remains unknown. In addition, the possibility that the previous observations have arisen from between-group differences in response bias, rather than perceptual differences, has not been addressed. Participants completed simultaneity judgements of audiovisual speech stimuli across a range of stimulus-onset asynchronies. Response times and accuracy data were fitted to a drift-diffusion model so that the drift rate (a measure of processing efficiency) and starting point (response bias) could be estimated. In Experiment 1, we tested a sample of non-autistic adults who completed the Autism Quotient questionnaire. Autism Quotient score was not correlated with either drift rate or response bias, nor were there between-group differences when splitting based on the first and third quantiles of scores. In Experiment 2, we compared the performance of autistic with a group of non-autistic adults. There were no between-group differences in either drift rate or starting point. The results of this study do not support the previous suggestion that autistic people have an extended temporal binding window for audiovisual speech. In addition, exploratory analysis revealed that operationalising the temporal binding window in different ways influenced whether a group difference was observed, which is an important consideration for future work.

## Introduction

Everyday perception involves the integration of signals received via different senses to create a representation of stimuli. It has been proposed that there is a *temporal binding window* of multisensory integration to account for the different physical and neural transmission rates of these sensory signals. The temporal binding window refers to the range of stimulus-onset asynchronies (SOAs) over which asynchronous crossmodal stimuli are integrated. This was initially observed in single-cell recordings in the cat superior colliculus (Meredith et al., 1987) and then across a range of experimental paradigms in humans (see Zhou et al., 2020 for a systematic review). This has been assessed using psychophysical procedures investigating acuity for event timing. The most common methods are the simultaneity judgement (SJ) task (Stone et al., 2001; Zampini et al., 2005), in which the participant is asked whether crossmodal stimuli presented at a range of temporal separations (SOAs) occurred at the same time, and temporal order judgement tasks (Hirsh & Sherrick, 1961; Zampini et al., 2003), where the participant is asked to discriminate the order of crossmodal stimuli presented at different SOAs. Another class of widely used experimental procedures indirectly assesses the temporal relationship between the senses by investigating the range of SOAs over which a stimulus impacts on the perception of another stimulus in a different sensory modality. For instance, these studies use the sound-induced flash illusion, in which a single flash presented with two beeps is misperceived as two flashes on many trials (see Hirst et al., 2020), or the McGurk effect, in which an incongruent visual speech stimulus can influence the perception of auditory speech (e.g., Munhall et al., 1996; van Wassenhove et al., 2007). The temporal binding window is believed to gradually contract across childhood development. Young children show worse temporal acuity on audiovisual SJ tasks compared with adults and this difference diminishes with increasing age (Chen et al., 2016; Hillock et al., 2011; Hillock-Dunn & Wallace, 2012; Kaganovich, 2016; Noel et al., 2016). The maturation of the temporal binding window is believed to be a key component in the development of sensory systems and to be impacted in forms of neurodivergence, such as autism (Robertson & Baron-Cohen, 2017; Stevenson et al., 2015).

Autism is a neurotype which shapes how a person processes information. Most autistic people experience differences in how common sensory stimuli are processed, in comparison with non-autistic people (Ben-Sasson et al., 2009; Kirby et al., 2022). These sensory differences include over- and under-responsiveness to stimuli, plus sensory-seeking and sensory-avoiding behaviours. These sensory differences are present throughout the lifespan (Crane et al., 2009; Tavassoli et al., 2014) and can be disabling in many contexts. Over the past 20 years, researchers have explored multisensory processing as a possible mechanism underlying autistic sensory differences using psychophysical and physiological experiments (Feldman et al., 2018) with a particular focus on the temporal binding window. Reduced connectivity in the posterior superior temporal cortex, a brain region associated with audiovisual speech (R. A. Stevenson et al., 2015) has been highlighted as a candidate neural mechanism. This reduced connectivity could lead to reduced temporal acuity of audiovisual signals and thus an extended temporal binding window. Studies which have compared measures of audiovisual temporal binding windows between autistic and non-autistic participants are described in Table 1 and reviewed in detail below.

**Table 1.** Studies which have compared autistic and non-autistic temporal binding window for audiovisual stimuli.

Study	Stimuli	Task type	Age	Measure of temporal binding window width	Between-group difference
Ainsworth & Bertone (2022)	Simple	Explicit (SJ)	Children	Standard deviation of Gaussian function	x
Foss-Feig et al. (2010)	Simple	Implicit (flash–beep illusion)	Children	Continuous span of SOAs where illusion reported significantly more than baseline	NA
Kwakye et al. (2011)	Simple	Implicit (temporal ventriloquism)	Children	Continuous span of SOAs with performance gains compared with baseline	NA
Noel et al. (2018)	Speech	Explicit (SJ)	Children	Standard deviation of Gaussian function	✓
R. A. Stevenson et al. (2014)	Simple, Complex, Speech	Explicit (SJ)	Children	Sum of the 75% point of two sigmoid functions	Simple: x Complex: x Speech: ✓
R. A. Stevenson et al. (2018)	Simple	Explicit (TOJ)	Children	Sum of the 75%–25% point of two sigmoid functions	x
Smith et al. (2017)	Complex Speech	Explicit (SJ)	Children	Sum of the 75% point of two sigmoid functions	x
Woynaroski et al. (2013)	Speech	Implicit (McGurk)	Children	75% point of sigmoid function	✓
Zhou et al. (2022)	Simple, Speech	Explicit (SJ)	Children	Standard deviation of Gaussian function	Simple: ✓ Speech: ✓
Borgolte et al. (2021)	Simple	Implicit (flash–beep illusion)	Adults	Continuous span of SOAs where illusion reported significantly more than baseline	x
De Boer-Schellekens, Eussen et al. (2013)	Simple Complex Speech	Explicit (TOJ)	Adults^a^	0.675/β of linear function	✓
de Boer-Schellekens, Keetels et al. (2013b)	Simple	Implicit (temporal ventriloquism)	Adults^a^	0.675/β of linear function	x
Poole et al. (2017)	Simple	Explicit (TOJ)	Adults	0.675/β of cumulative Gaussian function	x
Weiland et al. (2022)	Simple	Explicit (SJ)	Adults	Standard deviation of Gaussian function	x

SJ: simultaneity judgement; SOA: stimulus-onset asynchronies.

a Age: adults (>16 years).

Simple stimuli are low level, such as a flash and beep, complex stimuli are non-speech video recordings (hand claps, tools, or bouncing balls). Age: children (0–18 years) and adults (>18 years). Between-group difference: statistically significant between-group differences in comparison to the measure of temporal binding window; ✓ = increased in autistic group, x = no statistically significant difference, and NA = no between-group statistical test; TOJ = Temporal Order Judgement.

In the first study to empirically investigate temporal binding windows (Foss-Feig et al., 2010), autistic and non-autistic children completed a version of the sound-induced flash illusion where the SOA of the sound was systematically varied (0–500 ms in 100 ms increments). The authors defined the temporal binding window as the consecutive span of SOAs over which the illusory percept was reported significantly more than a one-flash one-beep condition, in each group. By this measure, the autistic group had a temporal binding window spanning 600 ms, whereas the controls had a window of 300 ms. Follow-up studies from the same group described auditory effects on visual perception across a wider range of SOAs in autistic children on visual temporal order judgements (temporal ventriloquism; Kwakye et al., 2011) and McGurk speech stimuli (Woynaroski et al., 2013). Corroborating evidence has been observed in studies using event timing tasks which have revealed reduced audiovisual temporal acuity in autistic participants. De Boer-Schellekens, Eussen et al. (2013) asked young adults (aged 16–22 years) to judge the temporal order of simple flash–beep and socially relevant stimuli (a hand clap and speech). Responses were fitted to a psychometric function to calculate the just noticeable difference, which was increased in autistic compared to non-autistic. Noel et al. (2018) used an SJ task on the auditory and visual components of speech syllables and found that the standard deviation of a fitted Gaussian function was larger in autistic compared with non-autistic children. Similar findings were recently observed by Zhou et al. (2022), who also observed the same effect for flash–beep stimuli.

These studies provide evidence from a range of tasks that the temporal binding window is extended in autism. Indeed, in a meta-analysis of six studies, an increased temporal binding window in autistic compared with non-autistic participants had a large average effect size (*g* = 0.86, 95% CI = [0.52, 1.15]; Zhou et al., 2018). It is also worth noting that extended temporal binding windows have also been observed in dyslexia (Francisco et al., 2017) and schizophrenia (Zvyagintsev et al., 2017), which are other forms of neurodivergence in which sensory differences are experienced. In addition to impacting on basic sensory function, the extended temporal binding window in autism is proposed to have a cascading effect on speech perception. Support for this has been claimed from the observation that autistic participants’ temporal binding windows are negatively correlated with the tendency to report the illusory stimuli on the McGurk task (R. A. Stevenson et al., 2014). In addition, children with a history of speech and language impairments are less accurate on an audiovisual SJ task than neurotypical children (Kaganovich, 2017; Kaganovich et al., 2014). Furthermore, in studies of non-autistic people, measures of performance on SJ tasks are correlated with scores on the Autism Quotient questionnaire (Donohue et al., 2012; Van Laarhoven et al., 2019. See Kawakami et al., 2020 for contradictory findings). Studies in non-autistic participants have shown that it is possible to narrow participants’ temporal binding window with feedback training (Horsfall et al., 2021; McGovern et al., 2022; Powers et al., 2009, 2012). Improvements on a speech in noise paradigm have also been observed following this training (Zerr et al., 2019), suggesting transfer effects to speech perception. This training has been highlighted as an approach which could be extended to autistic participants (R. A. Stevenson et al., 2015).

However, there are studies which have provided contradictory evidence regarding the extended temporal binding window for audiovisual stimuli in autism. Studies in children have observed no difference in acuity on a temporal order judgement tasks using flash–beep stimuli (A. R. Stevenson et al., 2018) and on an SJ task using speech stimuli (Smith et al., 2017). A recent study of SJs to flash–beep stimuli in children and adolescents found no overall group difference between the autistic and non-autistic group (Ainsworth & Bertone, 2022). There was an interaction between age and group, such that autistic children (aged <13 years) tended to have a wider temporal binding window than the non-autistic group, but there was no group difference for adolescents (aged 13–18 years). In addition, no correlation between temporal binding window width and scores on a sensory questionnaire was observed. A number of studies in adults have not observed differences in audiovisual temporal binding window width between autistic and non-autistic participants. No differences in temporal acuity were observed on temporal order judgements for flash–beep stimuli (Poole et al., 2017). Similarly, de Boer-Schellekens, Keetels et al. (2013b) did not observe between-group differences on a similar temporal order judgement task to that used in Kwakye et al. (2011) in a sample of young adults (aged 15–24 years). A recent study attempted to replicate Foss-Feig et al.’s (2010) study in an adult sample while also measuring event-related potentials (Borgolte et al., 2021). There were no differences between the groups in the behavioural data. The autistic participants showed an increased P2 amplitude, which is believed to be a neural correlate of multisensory processing. Another recent study, making use of a larger sample, observed no differences between groups of adults on an SJ task using flash–beep stimuli (Weiland et al., 2022).

### Some outstanding issues in the study of the temporal binding window in autism

There are issues to be considered which need to be resolved in the studies of temporal binding window to advance the understanding of mechanisms underlying sensory differences in autism. First, there are some inconsistencies in the findings in studies to date. The extended temporal binding window in autism may be dependent on whether speech stimuli are used (R. A. Stevenson et al., 2014, 2018). In addition, there is limited evidence of an extended temporal binding window in adults. It may be that the temporal binding window matures across a delayed trajectory in autism and is no different to non-autistic people by adulthood (Casassus et al., 2019). However, to date, only one study has investigated temporal acuity to audiovisual speech stimuli in autistic adults (de Boer-Schellekens, Keetels et al., 2013).

Second, as can be noted from the above discussion, a range of explicit and indirect methods has been used. The way in which the temporal binding window has been operationalised has also been varied. Studies using indirect methods have calculated the temporal binding window as the consecutive range of SOA over which illusory percepts were reported statistically significantly more often than baseline conditions (without correction for familywise error; Foss-Feig et al., 2011; Kwakye et al., 2011). Studies using event timing tasks have fitted participant data to a psychometric function to extract measures of acuity. As has been noted elsewhere (García-Pérez & Alcalá-Quintana, 2012; Yarrow et al., 2011; Yarrow & Roseboom, 2017), fitting data to a psychometric function involves making assumptions about the data and underlying psychological processes which are not always justified by the authors. The SJ task used in the studies described above requires a yes/no response asking the participant to detect a signal (the simultaneous stimuli) on each trial. However, the task is frequently treated as a discrimination task with researchers fitting two separate monotonic functions to the data (for auditory leading and lagging the visual stimuli) and calculating the temporal binding window as the sum of the distance between the 70% and 50% point extracted from each function (Smith et al., 2017; Van Laarhoven et al., 2019; Wallace & Stevenson, 2014). Note that when these two functions did not cross, researchers refit using new starting values, although the number of participants this applies to is not reported. An alternative approach has been to fit all responses to a Gaussian function to calculate the standard deviation as a measure of the temporal binding window (see Noel et al., 2018).

Third, it is also important to discount the role of non-perceptual, decision processes in the observed between-group differences. The differences in the temporal binding window have been characterised as the consequence of multisensory integration, a perceptual effect. However, the possibility that differences in response bias (the tendency to favour one response over another, regardless of the stimulus) may have impacted on the results of many studies has not been adequately addressed. For instance, in the Foss-Feig et al.’s study, there was a statistically significant effect of “group” (using a mixed analysis of variance [ANOVA]), suggesting that the autistic group was more likely to respond “two” whenever they heard double beeps regardless of SOA. Similarly, in the Woynaroski et al.’s study, the autistic group reported that the McGurk percept reported more often across all the SOAs which could be interpreted as a response criterion effect (autistic participants being more likely to use the “da” response). Decision processes can also impact the measure of temporal acuity on explicit tasks (see Yarrow et al., 2011). Autistic participants could have a more liberal response bias for responding “yes” on SJ tasks. In the study which used Bayesian decision theory to model performance on the SJ task (Noel et al., 2018), it was found that the autistic group had an increased prior to report common cause, which could plausibly be interpreted as a consequence of a more liberal response bias. To date, no studies of the temporal binding window in autism have appropriately dealt with the possible differences in response bias.

### Study aims and hypothesis

In this study, we aimed to replicate the previous observation of reduced audiovisual temporal acuity in autism while addressing some of the issues described above. We used audiovisual speech stimuli in an adult sample, modelling the study on the speech condition used in R. A. Stevenson et al. (2014) who reported a group difference in children. Evidence for an extended temporal binding window in autistic participants has been more consistently observed in studies in children using audiovisual speech stimuli, but there is only one study to date which has used these stimuli in autistic adults. We extended previous work by analysing participant responses using a drift-diffusion model (Ratcliff, 1978; Ratcliff & McKoon, 2008). In brief, the drift-diffusion model is a computational model of the participant’s decision process on binary response tasks. Each response option (“synchronous” or “asynchronous” on an SJ task) is characterised as a boundary. A process of noisy evidence accumulation takes place once the stimulus has been encoded until a boundary has been reached which triggers that response. There are many benefits of using the diffusion model over standard measurement of accuracy and response times (Forstmann et al., 2016). The parameters which can be estimated from the model have psychologically meaningful interpretations. The drift rate (*v*) determines the rate at which the evidence accumulation process takes place. Increased *v* is an index of a more efficient sensory evidence accumulation process. The boundary separation (β) is the distance between the boundaries, which is set before the stimulus arrives. The boundary separation is a measure of speed/accuracy trade-offs; larger values mean that the participant is more conservative in their decision-making. Non-decision time (*T_er_*) is the time taken for non-decision-making aspects of the response, incorporating the encoding of the stimuli and the execution of the movement. Finally, the starting point of the diffusion process (*zr*) is a measure of response bias. For instance, where the starting point is closer to the “simultaneous” boundary, the diffusion process will reach that boundary more often and rapidly than the “asynchronous” boundary. The diffusion model combines participant accuracy and response times in a principled way (Hedge et al., 2018) while increasing statistical power (Ratcliff, Thompson et al., 2016; Stafford et al., 2020).

There is a long history of the diffusion model in experimental psychology (Heitz, 2014; Ratcliff, Smith et al., 2016; Voss et al., 2013), but in recent years, it has been valuable in reinterpreting autistic performance on a number of tasks. To date, this has included the perception of orientation (Pirrone et al., 2017, 2020), motion (Manning et al., 2022), and faces (Powell et al., 2019), crossmodal selective attention (Poole et al., 2021), and inhibitory control (Karalunas et al., 2018). Importantly, modelling the data in this way allows perceptual (*v*) to be separated from decision-making (*zr*, β) and non-decision components (*T_er_*) of the response. For instance, Pirrone et al. (2017) found that autistic participants responded more slowly (but not less accurately) than non-autistic participants on an orientation judgement task, which could lead to the interpretation that acuity to orientation is reduced in autistic participants. Diffusion modelling (Wagenmakers et al., 2007) revealed that there were between-group differences in the boundary separation and non-decision time parameters, but not drift rate. This suggested that the response time differences were driven by more cautious responding by the autistic group and delay in encoding of the stimuli/executing the response rather than perceptual differences. In the context of the present investigation, if autistic participants have an extended temporal binding window compared with non-autistic participants, then the drift rate should be reduced relative to controls as the autistic participants will have difficulties parsing the stimuli on more trials. However, if the differences are related to response bias, then autistic participants will have a starting point closer to the “simultaneous” boundary. We examined these hypotheses across two experiments. In Experiment 1, we assessed the relationship between diffusion model parameters and Autism Quotient (AQ-50) scores in a sample of non-autistic adults. In Experiment 2, we explored differences in parameter estimates between autistic and non-autistic participants.

## Experiment 1

In Experiment 1, we had two hypotheses: if the temporal binding window is extended in individuals with increased scores on the AQ-50 questionnaire (see Donohue et al., 2012; Van Laarhoven et al., 2019), then the drift rate parameter (*v*) should be reduced with increasing AQ-50 score (Hypothesis 1; i.e., negative correlation between *v* and AQ-50). However, if this previously observed effect was driven by the differences in response bias which are associated with AQ-50 score, then the response bias parameter (*zr*) would be associated with increased scores on the AQ-50 (Hypothesis 2; i.e., a positive or negative correlation between AQ-50 and *zr*).

## Method

The methods and analysis used in this study were preregistered (https://osf.io/hknfq).

### Participants

Overall, 146 participants (76 males, 69 females, and 1 not disclosed) with a mean age of 28.76 (*SD* = 7.27) years were recruited to the study through Prolific Academic (https://www.prolific.co/). Following the removal of participants according to our preregistered criteria (see “Data Analysis” section), the final sample was 131 participants (72 males, 58 females, 1 not disclosed) with a mean age of 28.62 (*SD* = 7.06)  years. The study lasted approximately 35 min, and participants received reimbursement of £5 for taking part. The University of Manchester Ethics Committee approved the study. All participants completed an online consent form before taking part.

### Materials

The stimuli were videos developed from the congruent “ba” McGurk stimulus used in Basu Mallick et al.’s (2015) study (downloaded from https://openwetware.org/wiki/Beauchamp:Stimuli, video 4.6). First, background sound was removed using Audacity (https://www.audacityteam.org/). This video was used as the 0-ms SOA stimulus. Second, the relative timing of the auditory aspect of the video was edited using ShotCut (https://shotcut.org/) to create 18 further videos at SOAs of ± 594, 528, 396, 297, 264, 198, 165, 99, and 66 ms (where the auditory stimuli led and lagged for negative and positive SOA, respectively). These SOA were selected to be like those used in R. A. Stevenson et al.’s (2014) study with the precise timing constrained by the number of frames (30 fps) between the audio and visual stimuli. We added extra SOAs (±594, 528, and 396 ms) to ensure that there were trials in which all participants could easily complete the task to avoid loss of motivation. Third, 10 further videos were prepared as catch trials. The visual component of the stimulus was the same, but the audio was instead a person saying a number (e.g., “ten”).

The AQ-50 (Baron-Cohen et al., 2001) is a 50-item Likert-type response-type questionnaire relating to strengths, weaknesses, and preferences in daily life. The AQ-50 was designed to measure “autistic traits,” although the idea that any personality traits can be considered characteristic of autism has been challenged (Chown, 2019; Sasson & Bottema-Beutel, 2022). The AQ-50 is typically shown to have reasonable internal and test–retest reliability (Stevenson & Hart, 2017). In the current study, Cronbach’s alpha = .87, 95% CI = [.82, .89]. Items on the questionnaire are divided into domains aiming to measure social ability (“I find it hard to make new friends”), communication (“Other people frequently tell me that what I have said is impolite”), imagination (“I find it difficult to imagine what it would be like to be someone else”), attention to detail (“I often notice small sounds when others do not”), and attention switching (“I frequently get absorbed in one thing”). The underlying factor structure does not map onto these domains, although social skill and attention to detail are commonly identified as a major component on the AQ-50 (English et al., 2020; Hoekstra et al., 2008; Russell-Smith et al., 2011).

### Procedure

Participants indicated if they thought the auditory and visual stimuli in each video were “in time” (synchronous) or not (asynchronous). Participants were asked to respond accurately, but promptly. On each trial, participants were first presented with a fixation cross with the duration of the fixation randomly selected on each trial with *M* = 600 ms and range = 100 ms. The video was then presented with the response options (Yes = f, No = j) visible on screen. After participants responded, they were presented with a screen indicating that they could commence the next trial when ready. An onscreen progress bar indicated how far through that block they were. On catch trials, participants were presented with a screen in which they were asked to type out which number was said. Participants were asked to wear headphones during the task.

Participants completed 10 blocks of trials with each SOA presented twice and a single catch trial in a random order. This gave a total of 20 repetitions of each SOA and 400 experimental trials in total.

After completing the SJ, participants completed the AQ-50. Two catch questions (see Buchan & Schofield, 2018) were included to identify participants not reading the questions.

The experiment was controlled using Gorilla (Anwyl-Irvine et al., 2019). Participants were not supervised by a researcher while completing the task which means we did not control where participants were, or what hardware they used when completing the task. At the end of the study, we asked participants to respond truthfully as to whether they wore headphones, 97.26% of participants answered yes.

The materials are available on the Gorilla Open repository (https://app.gorilla.sc/openmaterials/421353).

### Data analysis

#### Exclusion of participants

There were a set of preregistered criteria for identifying participants who did not understand the task or were low-effort responders. One participant was removed for performing <70% accuracy on the catch trials. However, ten participants were removed who responded “Yes” more frequently on the ±528-ms SOA compared with the 0-ms SOA, one participant was removed who failed both catch questions on the AQ-50.

#### Data preparation

**Figure 1.**
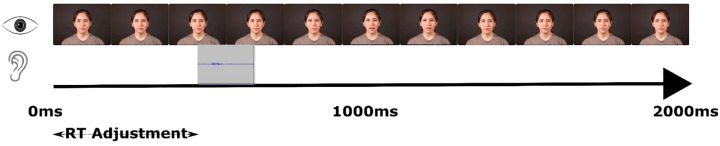
Schematic of the visual and auditory stimuli used in a trial. The response time adjustment was from the beginning of the video to the onset of the auditory stimuli, or lip movement (began at 891 ms) whichever came first for that SOA. Video stills from https://openwetware.org/wiki/Beauchamp: Stimuli available under a creative commons licence (https://creativecommons.org/publicdomain/zero/1.0/).

Data preparation was conducted in R using the following packages: readr (Wickham et al., 2021), reshape2 (Wickham, 2007), VarHandle (Mehrad, 2020), stringr (Wickham, 2019), janitor (Firke, 2021), and tidyverse (Wickham et al., 2019). First, response times were corrected by deducting the time from the beginning of the video to the onset of the stimuli. We decided to adjust the videos in this way as we expected that the accuracy in estimates of drift rate would be compromised for overly long response times (Voss et al., 2015), leading to an increased false-negative rate. Response times were adjusted to the beginning of the first part of the stimuli (visual or auditory; see Figure 1). The response times were adjusted as follows: For all positive SOA, −99, −66, and 0 ms RT = RT − 891 ms. For −594 ms, RT = RT − 396 ms; for −528 ms, RT = RT − 462 ms; for −396 ms, RT = RT − 594 ms; for −297 ms, RT = RT − 693 ms; for −264 ms, RT = RT − 726 ms; for −198 ms, RT – RT − 792 ms; and for −165 ms, RT = RT − 825 ms.

Second, in order that only responses to asynchronous stimuli were modelled, the 0-ms SOA was excluded. Third, to reduce the impact of anticipations and misses on model fitting, individual participant response time outliers were trimmed using the Van Selst and Jolicoeur (1994) non-recursive procedure across correct and error responses using the trimr package (Grange, 2018). This led to 3.31% of trials from the total dataset being trimmed. Following trimming of individual response times, the number of trials for each participant was ⩾ 342.

#### Model fitting

**Figure 2.**
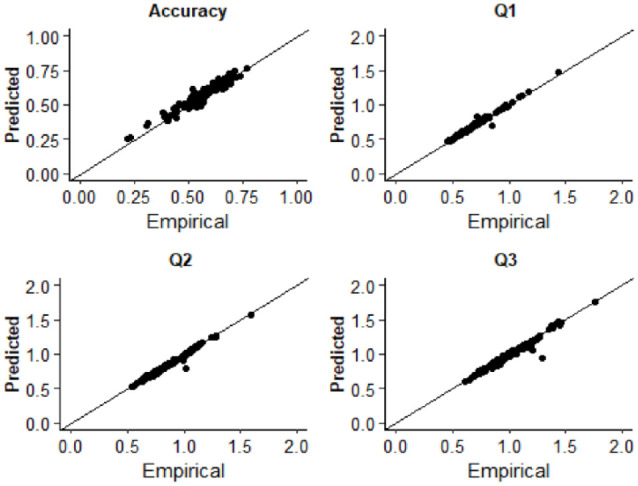
QQ plots displaying the empirical and predicted accuracy and the first (Q1), second (Q2), and third (Q3) quantiles of the response time distributions in Experiment 1. Data points are estimates for individual participants.

Response times for asynchronous (correct) and synchronous (error) responses were used to estimate diffusion model parameters. Fitting was conducted using the Kolmogorov–Smirnov procedure as implemented using fast-dm-30 (Voss et al., 2015). Four parameters were left free to vary in the fitting procedure: drift rate (*v*), boundary separation (α), non-decision time (*t*_0_), and starting point (*z*). Intertrial variability of starting point and drift rate was set to zero. To assess the extent to which the model was an appropriate fit of the empirical data, we used construct samples in fast-dm-30 to sample 1,000 trials from the parameter estimates for each participant. The proportion accuracy and first, second, and third quantiles of the response time distribution were calculated and plotted as a function of the empirical data in QQ-Plots (see Figure 2). The empirical and sampled data fall along the x = y line suggesting that the model provided a good fit to the empirical data. Additionally, we plotted both empirical and sampled cumulative density functions for individual participants response time distributions which are available on the study OSF page (https://osf.io/3fjbs/).

### Analysis

The following R packages were used for data analysis and plotting: cowplot (Wilke, 2020), effsize (Torchianio, 2020), and BayesFactor (Morey & Rouder, 2019). To prevent the impact of extreme values on analysis, group-level outliers that were three absolute median deviations from the group median were identified using the Routliers package (Delacre & Klein, 2019) and were winsorised. To test Hypothesis 1, a Pearson correlation coefficient was calculated to assess the relationship between total score on the AQ-50 and *v* (drift rate). In addition, we compared *v* between participants in the first and third quantiles of AQ-50 scores using a between-groups *t*-test. To test Hypothesis 2, we ran the same analysis for *zr* (response bias).

## Experiment 1 results

Raw, aggregate data and analysis code are available on the study OSF page (https://osf.io/3fjbs/)

**Figure 3.**
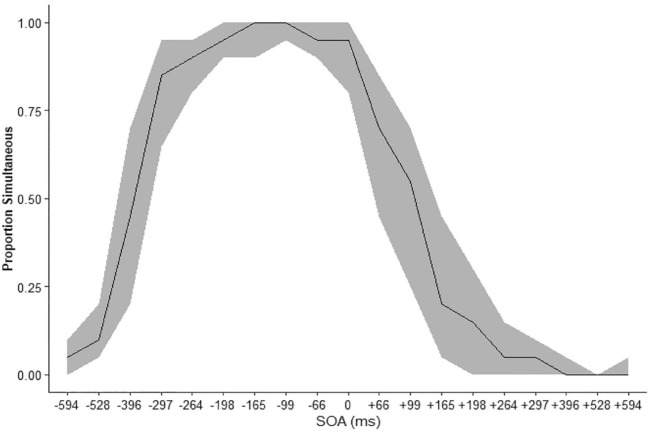
The median proportion simultaneous responses at each SOA smoothing represents the IQR. Sign indicated the auditory stimulus relative to the visual with auditory leading for negative SOA and lagging for positive SOA.

The proportion of trials in which participants made simultaneous judgements Figure 3. The peak of simultaneous responses was shifted left, meaning that participants tended to report the stimuli as simultaneous when the auditory stimuli preceded the visual.

**Figure 4.**
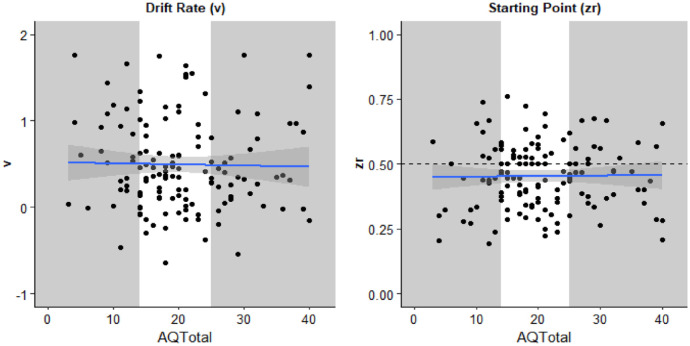
Parameters estimated from the diffusion model drift rate (*v*, left) and starting point (*zr*, right) as a function of participants total score on the AQ-50. Each individual is represented by a data point. The solid line is the best fitted line with smoothing. The grey sections highlight the individuals in the first and third quantiles of AQ-50 total whose parameter estimates were compared using *t*-tests.

The mean of participants total score on the AQ-50 *=* *20.30 (SD* *=* 8.39). The mean estimates of the diffusion model parameters were *v* = 0.50 (*SD* = 0.53) and *zr* = .453 (*SD* = 0.124). Model parameters for each participant are displayed in Figure 4 as a function of that participants’ score on the AQ-50.

Pearson correlation coefficients were calculated to explore the relationship between the parameter estimates and participants total score on the AQ-50. There was no statistically significant correlation for either *v, r* (134) = −.020, *p* = .810, 95% CI = [−.188, .148], *BF*_10_ = 0.20, or *zr, r* (134) = .014, *p* = .872, 95% CI = [−.15, .18], *BF*_10_ = 0.20.

Second, participants in the first and third quantiles on the AQ-50 were compared using an independent-samples *t*-test. There was no significant difference for *v, t*(44.82) = 1.376, *p* = .176, mean difference = 0.18, 95% CI = [−0.09, 0.47], *d* = 0.37, 95% CI = [−0.16, 0.89], *BF*_10_ = 0.61, nor *zr, t*(39.35) = 0.78, *p* = .438, mean difference = 0.03, 95% CI = [−0.10, 0.05], *BF*_10_ = 0.35.

### Exploratory analysis

**Figure 5.**
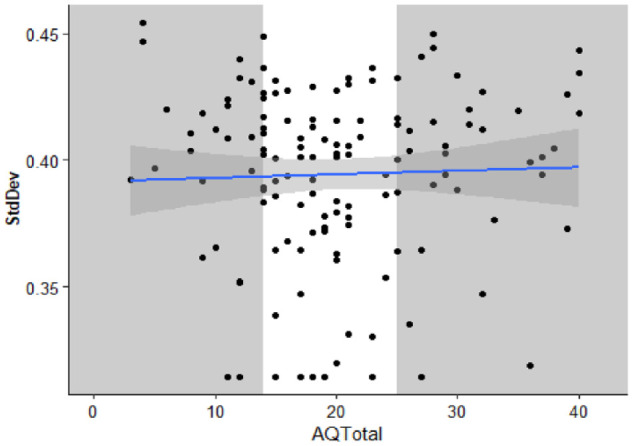
The standard deviation of a Gaussian function fitted to participant proportion “Simultaneous” responses as a function of total AQ-50 score. Each individual is represented by a data point. The solid line is the best fitted line with smoothing. The grey sections highlight the individuals in the first and third quantiles of AQ-50 total whose parameter estimates were compared using *t*-tests.

To aid comparison with previous work, we conducted further exploratory, non-preregistered analysis. The standard deviation of a Gaussian function was estimated as a measure of the temporal binding window (similar to the method used in Donohue et al., 2012; Noel et al., 2018). Each participant’s proportion of “simultaneous” responses, here including the 0-ms SOA, was fitted to a Gaussian function using the fitdistrplus package (Delignette-Muller & Dutang, 2015). The mean standard deviation = 0.39 (*SD* = 0.04). Individual participant standard deviations are displayed in Figure 5. Outliers were winsorised as described for the diffusion model analysis.

A Pearson correlation coefficient was calculated to explore the relationship between participant standard deviation and total score on the AQ-50. There was no statistically significant correlation between participant standard deviation and total score on the AQ-50, *r* (134) = .03, *p* = .702, 95% CI = [−.14, .20], *BF*_10_ = 0.21, nor significant difference between the upper and lower quartiles on the AQ-50, *t*(43.25) = −0.28, *p* = .781, mean difference < −0.01, 95% CI = [−0.02, 0.01], *d* = −0.07, 95% CI = [−0.59, 0.44], *BF*_10_ = 0.27.

As a further exploratory analysis, we have plotted each subscale on the AQ-50 as a function of the dependent variables from the SJ task (see supplementary materials https://osf.io/w3bdf). There appears to be no meaningful relationship between any of the measures and these subscales.

## Experiment 2 introduction

In Experiment 2, autistic and non-autistic participants completed the online SJ task described in Experiment 1. We had two hypotheses: (1) if autistic people have an extended temporal binding window in comparison to non-autistic people, then *v* would be expected to be decreased in comparison with the non-autistic group; (2) if previously observed differences were reflective of differences in response bias between the groups, then *zr* would differ between the groups

## Method

The method and analysis of Experiment 2 were preregistered (https://osf.io/b4dky)

### Participants

There were 31 autistic (13 females) and 30 non-autistic participants (12 females).^1^ Participants were recruited from databases at the University of Manchester, City, University of London and the University of Sheffield. All participants had previously participated in laboratory visits in these groups and had consented to having their contact details and some data retained for further studies. Participants were emailed a copy of the participant information sheet, and those who responded expressing an interest in taking part were sent the study link and unique ID. Participants who expressed an interest in taking part, but did not complete the study were emailed up to two times with prompts reminding them to take part. Once all eligible participants had been contacted and all prompts had been sent, data collection ended.

All participants had previously completed the Wechsler Abbreviated Scale of Adult Intelligence (WASI; Wechsler, 2008) during in-person laboratory visits. All autistic participants had a professional diagnosis of autism which had been confirmed when participating in previous research by showing a letter. Autistic participants had also completed module five of the Autism Diagnostic Observation Schedule (Lord et al., 2000) during previous in-person studies and had scored above the cut-offs for autism. The groups were well matched for age and Full-Scale IQ (FSIQ; see Table 2). The study procedures were approved by the University of Manchester Ethics Community. All participants completed an online consent form before taking part in the study. Participants received a £10 voucher as compensation for taking part in the study.

**Table 2.** Participant age and FSIQ measured on the WASI plus the effect size (*d*) of the difference between groups and variance ratio (*F*).

	Autistic, *M* (*SD*)	Non-Autistic, *M* (*SD*)	*d* [95% CI]	*F* [95% CI]
Age	32.80 (7.92)	34.90 (5.84)	0.29 [−0.22, 0.81]	1.84 [0.89,3.83]
FSIQ	118 (7.92)	113 (5.84)	0.36 [−0.16, 0.88]	0.83 [0.39, 1.73]

*SD*: standard deviation; CI: confidence interval.

### Materials and procedure

The study was conducted online, the materials and procedure of the simultaneity task was identical to Experiment 1, although in Experiment 2 participants were not asked to complete the AQ-50. When asked at the end of the experiment, 80.76% of the autistic group and 95% of the non-autistic group responded “yes” that they had worn headphones.

### Data analysis

**Figure 6.**
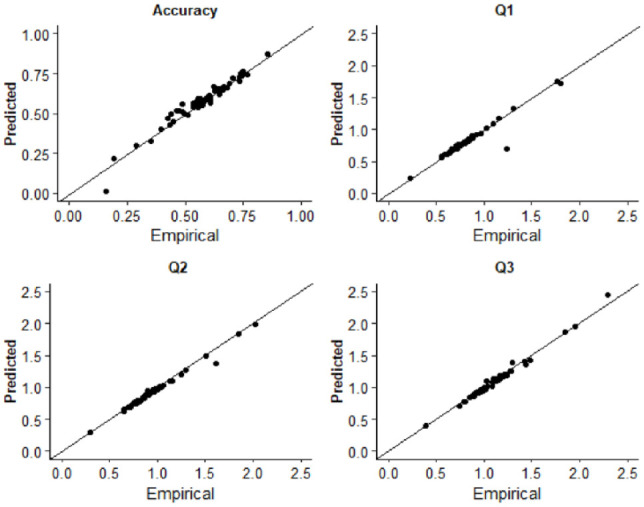
QQ plots displaying the empirical and predicted accuracy and the first (Q1), second (Q2), and third (Q3) quantiles of the response time distributions in Experiment 2. Data points are estimates for individual participants. Note that autistic and non-autistic participants are combined in this figure.

The same criteria were used for identifying participants who did not understand the task or were low-effort responders as Experiment 1. Two autistic and two neurotypical participants were removed who produced “Yes” responses more frequently to the ± 528-ms SOA compared with the 0 ms. Data preparation, model fitting, and assessment of model fits were identical to that described in Experiment 1. RT trimming removed 3.35% of all trials in the autistic group and 3.49% for the non-autistic group. As can be seen in Figure 6, the predicted and empirical data generally fall along the x = y line, suggesting the model provided a good fit of the data for all participants (see the study OSF page for individual participant predicted and empirical reaction time cumulative density functions).

A one-tailed, between-samples *t*-test was used to test Hypothesis 1 that estimates of *v* would be lower for the autistic compared with the non-autistic group. A two-tailed between-samples *t*-test was used to test Hypothesis 2 that estimates of *z* would be different between the autistic and non-autistic groups.

## Experiment 2 results

Raw, aggregate data, and analysis code are available on the study OSF page (https://osf.io/bmzuh/)

**Figure 7.**
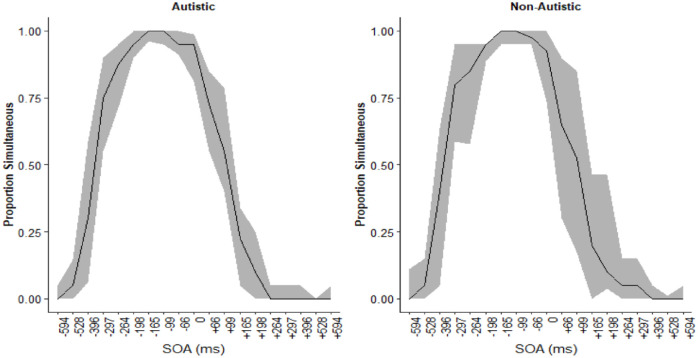
Proportion of simultaneous judgements at each SOA for the autistic (left) and non-autistic (right) groups. The black line represents the median proportion simultaneous judgements and the smoothing is the interquartile range. Polarity of the SOA reflects the timing of the auditory stimuli relative to the visual.

Accuracy and parameter estimates were similar between the groups. The proportion of trials in which participants made simultaneous judgements is displayed in Figure 7. Participants in both groups tended to report simultaneously more often for negative SOA (auditory leading trials).

**Figure 8.**
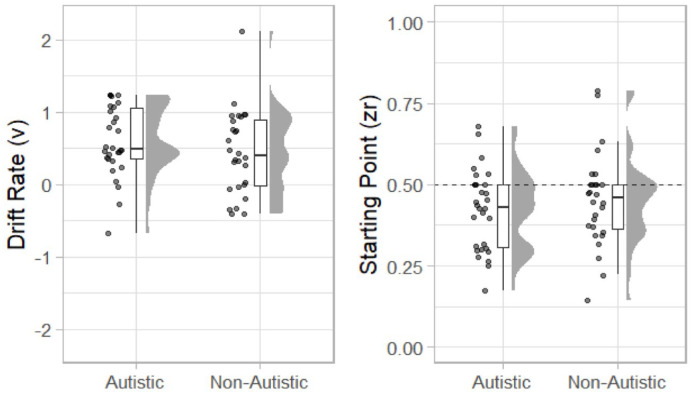
Raincloud plots displaying autistic and non-autistic participants drift rate (*v*; left) and starting point (*zr*; right).

Estimates of drift rate (*v*) and starting point (*zr*) are displayed in Figure 8. The mean value of *v* for the autistic group was 0.603 (*SD* = 0.49) and for the non-autistic group was 0.45 (*SD* = 0.58). A one-sided (non-autistic group *v* > autistic group) between-participants *t*-test was not statistically significant, *t*(56) = 1.11, *p* = .864, mean difference = −0.16, 95% CI = [INF, 0.39], *d* = 0.29, 95% CI = [−0.24, 0.82], *BF*_10_ = 0.14.

The mean value of *zr* for the autistic group was 0.422 (*SD* = 0.123) and for the non-autistic group 0.451 (*SD* = −0.143). As can be seen in Figure 8, participants in both groups tended to have a starting point <.50 suggesting that they were biased towards reporting the stimuli as simultaneous. A two-sided between-participants *t*-test was not statistically significant, *t*(56) = 0.85, *p* = .400, mean difference = −0.03, 95% CI = [−0.099, 0.040], *BF*_10_ = 0.36.

### Exploratory analysis

#### Standard deviation

**Figure 9.**
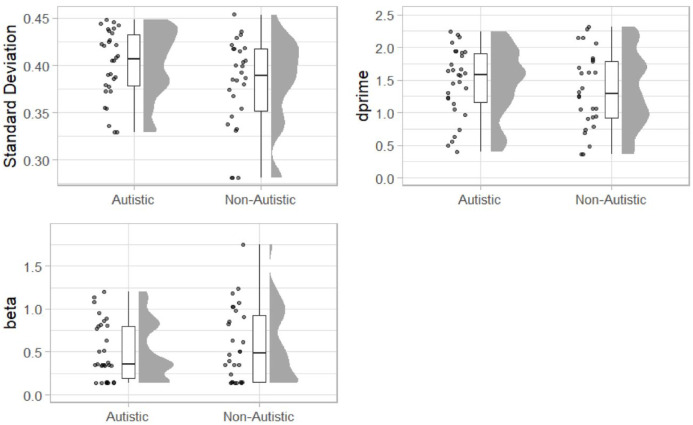
Raincloud plots displaying standard deviation extracted from a Gaussian function (top left) and signal detection parameters dprime (top right) and beta (bottom left) for the autistic and non-autistic groups.

To facilitate direct comparisons with previous work and inclusion in future meta-analysis, we conducted further exploratory, non-preregistered analysis. First, we calculated the standard deviation of a Gaussian function fitted to each participant’s “simultaneous” responses across the SOA (including the 0-ms SOA), as described in Experiment 1. Outliers were winsorised as described above for drift-diffusion model parameter estimates. Estimates of the standard deviation of the fitted function are displayed in Figure 9.

The standard deviation was increased in the autistic group (*M* = 0.40, *SD* = 0.04) compared with the non-autistics (*M* = 0.38, *SD* = 0.05). A one-sided between-groups *t*-test (autistic group standard deviation > non-autistic group) revealed that difference was statistically significant, *t*(56) = 1.85, *p* = .04, mean difference = 0.02, 95% CI = [<0.001, Inf], *d* = 0.48, 95% CI = [−0.05, 1.02], *BF*_10_ = 2.06.

#### Signal detection analysis

Finally, we used signal detection analysis to calculate a measure of sensitivity (*d*’) and bias (β). The 0-ms condition was treated as the signal-present condition with other SOA as signal absent. We calculated hits, misses, false alarms, and correct rejections for each participant. Signal detection parameters were then estimated using the *psycho* package (Makowski, 2018). Estimates of *d*’ (higher values = increased acuity) were increased in the autistic group (*M* = 1.45, *SD* = 0.53) compared with the non-autistic group (*M* = 1.34, *SD* = 0.58). A one-sided between-groups *t*-test was not statistically significant, *t*(56) = 0.82, *p* = .792, mean difference = 0.12, 95% CI = [−Inf, 0.36], *d* = 0.22, 95% CI = [−0.31, 0.74], *BF*_10_ = 0.54. Estimates of β (higher values = bias to respond ‘no’; more conservative responding) were lower in the autistic group (*M* = 0.509, *SD* = 0.34) compared with the non-autistic group (*M* = 0.586, *SD* = 0.43). A two-sided between-groups *t*-test was not statistically significant, *t*(56) = 0.76, *p* = .449, mean difference = −0.08, 95% CI = [−0.28, 0.13], *d* = −0.20, 95% CI = [−0.73, 0.33], *BF*_10_ = 0.34.

## Discussion

This study comprises two experiments investigating in Experiment 1, there was no correlation between total score on the AQ and a measure of processing efficiency (drift rate, *v*), nor response bias (starting point, *zr*) in a sample of non-autistic participants. Participants were also split by the first and third quantiles of AQ scores and these parameters were compared between groups, but there were no statistically significant differences. In Experiment 2, performance on the same task was compared between a sample of autistic and non-autistic participants. There were no significant differences between the groups in drift rate, nor starting point. The findings of Experiment 1 and 2 did not support our hypothesis and do not support previous work which has suggested that autistic people (Foss-Feig et al., 2010; Noel et al., 2018; R. A. Stevenson et al., 2014, Zhou et al., 2022) and non-autistic people with increased scores on the AQ (Donohue et al., 2012; van Laarhoven et al., 2019) have an extended temporal binding window. Furthermore, these findings do not support our secondary hypotheses that differences between the groups might be explained by differences in response bias. Overall, performance on the task was similar between autistic and non-autistic people, and total score on the AQ was not predictive of performance in non-autistic participants.

The current study is a valuable addition to the literature regarding the temporal binding window in autism. Following null findings when comparing performance for simple flash–beep stimuli (e.g., Poole et al., 2017; R. A. Stevenson et al., 2014), it has been suggested that autistic people have an extended temporal binding window when making judgements about speech stimuli specifically. The current study does not support this suggestion as drift rate, a measure of perceptual efficiency did not differ between groups. This contrasts with studies which have observed reduced acuity for event timing judgements about audiovisual speech stimuli in children (Noel et al., 2018; R. A. Stevenson et al., 2014) and in younger adults (de Boer-Schellekens, Eussen et al., 2013). This may support the suggestion that the narrowing of the temporal binding window across adolescent development (Hillock et al., 2011; Hillock-Dunn & Wallace, 2012) is delayed in autism, but reaches a similar level to non-autistic people in adulthood (Casassus et al., 2019). However, considering the current study in the context of other recent work (summarised in Table 1), we argue that there is limited evidence in support of an extended temporal binding window in autism.

Importantly, analytic decisions may impact on the measurement of between-group differences. In additional, non-preregistered analyses in Experiments 1 and 2, we fitted the accuracy data to a Gaussian function and used the standard deviation as a measure of temporal binding window, similar to previous work using AQ-50 in non-autistic participants (Donohue et al., 2012) and comparing autistic and non-autistic adults (Noel et al., 2018; R. A. Stevenson et al., 2014). In Experiment 1, there was no correlation with AQ-50 nor a difference in standard deviation between the first and third quantiles on the AQ-50. In Experiment 2, the standard deviation of the autistic group was increased compared with non-autistic participants, in line with the previous work. However, when using signal detection analysis, which also separated out response bias, there were no differences in sensitivity between the groups, similar to the preregistered analysis using the drift-diffusion model. Although there were no differences in metrics of response bias (starting point *zr* and beta), it is notable that there were no between-group differences when using approaches which partial out response bias and acuity, whereas a difference was observed for the analysis of standard deviation which did not take this into account. This suggests that how the temporal binding window is operationalised can influence whether a diminished performance is observed in an autistic sample. It is important to ascertain whether the observation of an extended temporal binding window in autism is robust to researchers’ analytic decisions. Indeed, theoretical models of performance on event timing tasks have long included decision-making (Sternberg & Knoll, 1973). The contribution of response bias to differences in performance in autistic children should be ruled out. As an extended audiovisual temporal binding window has been more commonly observed in studies using speech stimuli, another important outstanding question is whether differences are a consequence of altered timing process or in how autistic and non-autistic children attend to face stimuli (Guillon et al., 2014). This could be assessed in future work using eye movement recording while participants complete an SJ task.

### Limitations

In the current study, we fit participant data to a standard diffusion model. To our knowledge, this is the first time a diffusion model framework has been implemented in a study of simultaneity perception. In fitting this model, we made the assumption that a single, linear diffusion process would provide the best explanation of the decision-making process. There are variations of the drift-diffusion model with non-linear diffusion processes (for instance, the diffusion model for conflict; Ulrich et al., 2015). There is also an adaptation of the diffusion model for investigating interval timing (the time-adaptive opponent Poisson drift-diffusion model; Balcı & Simen, 2016). The standard diffusion model provided a good fit of the experimental data in both Experiments 1 and 2, but it is possible that an alternative model would have provided a superior fit of the data. Model comparison and development was beyond the scope of the current work, but this may be a valuable approach in the future. In particular, decision modelling could bring interval and event timing into a single framework.

The development and much of the data collection involved in this work took place while there were lockdown restrictions in the United Kingdom in the context of the COVID-19 pandemic. This influenced the study in two notable ways which may have impacted on the interpretations of the findings. First, the study was conducted online rather than in the laboratory, in contrast with all previous work investigating temporal binding windows in autism. The use of online testing in experimental psychology has exploded in popularity in recent years and most effects observed in the laboratory can be replicated in larger samples using online testing (Crump et al., 2013; Germine et al., 2012; Schidelko et al., 2021). In addition, the control of stimuli through Gorilla is favourable when compared with laboratory-based methods (Anwyl-Irvine et al., 2021). However, there is a loss of control of the conditions under which participants complete the task; particularly in unmoderated experiments such as the present work. Notably in the current study, there were a high proportion of simultaneous responses in the auditory leading SOA. Work comparing laboratory-based versus online testing has largely been focused on how similar (non-autistic) participant data collected online is to data collected in laboratory conditions (e.g., Germine et al., 2012). There has been less work focused on the participants’ experience of taking part. Autistic people often find travelling to and completing laboratory-based studies stressful (Gowen et al., 2019) and in this respect, online testing may be preferable for some autistic people. Second, in Experiment 2, the sample size was relatively low (although similar to previous laboratory-based studies). As highlighted in the preregistration document, our target sample size was larger, but only a small number of the participants who were contacted responded and eventually completed the study. This may relate to some combination of (a) lack of time and energy for participating in research during the pandemic; (b) not being motivated by the study; and (c) low interest in taking part in online research. However, despite the relatively small number of participants in Experiment 2, the study was adequately powered to detect the average effect (*g* = 0.86; Zhou et al., 2018) previously reported in a meta-analysis (a power calculation conducted using the pwr package revealed 17 participants in each group could detect an effect size of 0.86 with α = .05, β = .8 using a one-sided between groups *t*-test).

In summary, the current findings have not supported the previous observation regarding an extended audiovisual temporal binding window in autistic adults, nor in non-autistic adults who score highly on the AQ. It may be that the temporal binding window has matured to a similar level to non-autistic people by adulthood. However, our exploratory analysis has suggested that how the temporal binding window is operationalised may influence whether the effect is observed. Future work in autistic children should use reliable methods that discount the contribution of response strategy to better understand whether there is an extended temporal binding window in autism.

## Notes

1. Note the preregistered target sample size was 140 participants.
